# Tubulin Tyrosination Is Required for the Proper Organization and Pathfinding of the Growth Cone

**DOI:** 10.1371/journal.pone.0005405

**Published:** 2009-04-30

**Authors:** Séverine Marcos, Julie Moreau, Stéphanie Backer, Didier Job, Annie Andrieux, Evelyne Bloch-Gallego

**Affiliations:** 1 Institut Cochin, Université Paris Descartes, CNRS (UMR 8104), Paris, France; 2 Inserm, U567, Département Génétique et Développement, Paris, France; 3 Grenoble Institut des Neurosciences, Centre de Recherche Inserm U.836 – UJF-CEA-CHU, Bâtiment Edmond J. Safra, Université Joseph Fourier, Site Santé à La Tronche, Grenoble, France; Harvard University, United States of America

## Abstract

**Background:**

During development, neuronal growth cones integrate diffusible and contact guidance cues that are conveyed to both actin and microtubule (MT) cytoskeletons and ensure axon outgrowth and pathfinding. Although several post-translational modifications of tubulin have been identified and despite their strong conservation among species, their physiological roles during development, especially in the nervous sytem, are still poorly understood.

**Methodology/Findings:**

Here, we have dissected the role of a post-translational modification of the last amino acid of the α-tubulin on axonal growth by analyzing the phenotype of precerebellar neurons in *Tubulin tyrosin ligase* knock-out mice (*TTL* ^−/−^) through in vivo, ex vivo and in vitro analyses. *TTL* ^−/−^ neurons are devoid of tyrosinated tubulin. Their pathway shows defects in vivo, ex vivo, in hindbrains open-book preparations or in vitro, in a collagen matrix. Their axons still orient toward tropic cues, but they emit supernumerary branches and their growth cones are enlarged and exhibit an emission of mis-oriented filopodia. Further analysis of the *TTL* ^−/−^ growth cone intracellular organization also reveals that the respective localization of actin and MT filaments is disturbed, with a decrease in the distal accumulation of Myosin IIB, as well as a concomitant Rac1 over-activation in the hindbrain. Pharmacological inhibition of Rac1 over-activation in *TTL* ^−/−^ neurons can rescue Myosin IIB localization.

**Conclusions/Significance:**

In the growth cone, we propose that tubulin tyrosination takes part in the relative arrangement of actin and MT cytoskeletons, in the regulation of small GTPases activity, and consequently, in the proper morphogenesis, organization and pathfinding of the growth cone during development.

## Introduction

During embryonic development, axons have to make choices in order to reach their target in the nervous system. The neuronal growth cone is a dynamic structure at the tip of the axon that provides the traction force necessary for axon outgrowth and acts as a sensor. It explores the environment by integrating guidance cues that influence its direction and remodeling. These functions depend on the cytoskeleton reorganization and dynamics [1], [2]. Although the actin cytoskeleton has been proposed as a major target of guidance cues due to its peripheral location in the growth cone and its crucial role in extension and retraction of filopodia and lamellipodia when they encounter tropic cues, the microtubule (MT) cytoskeleton has also been involved in the guiding process through intracellular signaling pathways [3]. Changes in the organization of MTs were proposed to occur later in vivo, as a consequence of formation and disassembly of actin filaments. However, recent studies have revealed that MTs do not remain passively bundled in the growth cone center since a dynamic population of MTs composed of tyrosinated-tubulin is able to enter the actin-rich peripheral domain (P-domain) of the growth cone and to grow along actin filaments in filopodia [4].

We have studied the role of tubulin tyrosination, a post-translational modification of the tubulin that allows, after elimination of the C-terminal tyrosine of neo-synthesized α-tubulin by a carboxypeptidase, its re-addition by the tubulin tyrosine ligase (TTL). This post-translational modification leads to the presence of tyrosinated-tubulin (tyr-tubulin) at the dynamic plus-ends of MTs while older and more stable MTs are mainly composed of detyrosinated tubulin, called glutamylated-tubulin (glu-tubulin) [5], [6]. Despite the absence of functional links between the tyrosination state of MTs and their dynamics [7], it appears that dynamic MTs contain tyr-tubulin, whereas bundled MTs in the central domain of the growth cone (C-domain) are detyrosinated and more stable. In *TTL* knock-out mice (*TTL^−/−^*), detyrosinated tubulin is accumulated and mice die perinatally [8]. A previous analysis of *TTL* ^−/−^ mice has revealed that tyr-tubulin is involved in the control of proper neurite extensions of hippocampal neurons [8]. In addition, the recruitment of Clip-170, a plus-end tracking protein (+TIP protein) that binds to MTs, and of other MT +TIP proteins, which have a cytoskeleton-associated protein glycine-rich (CAP-Gly) MT binding domain, such as Clip115 and p150 Glued, is impaired in *TTL^−/−^* mice [9]. Thus, to better understand how the presence of the C-terminal tyrosine on α-tubulin influences neurite outgrowth, a deeper analysis of the intracellular organization of axons during outgrowth was necessary.

Precerebellar nuclei (PCN) provide an interesting model to study guidance cues. Indeed, PCN neurons, dorsally located in the rhombic lips, migrate ventrally through a tangential neurophilic migration [10], emitting first a leading process, the future axon, and then translocating their cell bodies in the leading process [11]. The floor plate is a source of both contact molecules and chemotropic factors, such as Netrin-1 that can influence both axon guidance and cell bodies migration of various PCN neurons [12], [13], [14] and act as an intermediate target for migrating PCN neurons.

Although admitted that axon growth and guidance depend on well-coordinated cytoskeletal dynamics, the direct characterization of specific cues remained a challenge. Only recently have small GTPases - in particular the Rho/Rho Kinase pathway and myosin II contractility - been altogether involved in regulation of microtubule behavior during neuronal growth [15]. It has also been reported that upon Myosin II inhibition, the movement of actin filaments and MTs immediately stopped and MTs unbundled in the gowth cone neck [16].

Here, we explore the role of tubulin re-tyrosination on growth cone pathfinding through the analysis of *TTL^−/−^* PCN neurons that are deprived of tyr-tubulin. We report that the pathfinding of *TTL^−/−^* axons is disturbed in the vicinity of the floor plate in vivo. Ex vivo, in hindbrain open-book preparations, growth cones are enlarged and exhibit a complex morphology with numerous mis-oriented filopodia, especially when reaching the floor plate. In vitro in a collagen matrix, axon outgrowth is decreased, although still oriented toward a local attractive Netrin-1 source. Supernumerary exploring branches also develop all along the axons. In addition, the cytoskeletal organization in the growth cone is disrupted since detyrosinated MTs abnormally enter the peripheral actin-rich domain and they exhibit a decreased local recruitment of Myosin IIB. These observations are concomitant and consistent with an increased activity of Rac1 small GTPase observed in *TTL^−/−^* hindbrains. The distal accumulation of Myosin IIB can be significantly rescued after pharmacological inhibition of Rac1 activity. Altogether, these results suggest that re-tyrosination at the MTs plus-ends is required for the formation and intracellular organization of a functional growth cone at the tip of the axon.

## Results

### In vivo, the migratory process develops properly in the absence of *TTL*, but inferior olivary fibers show an abnormal aspect in the vicinity of the floor plate

Newly synthesized α-tubulin carries a tyrosine residue on its C-terminal. Then the tyrosine residue is removed by a carboxypeptidase and TTL brings an additional tyrosine residue According to the level of transcription-translation in a cell, the amount in neo-synthesized tyr-tubulin varies. Thus, in *TTL^−/−^* cells, the amount of remaining tyr-tubulin varies according to cell types and tissues and their specific transcription-translation rate [9]. We first analyzed the tyr-tubulin content in PCN neurons with a specific antibody that only labels tyrosinated tubulin. In the absence of retyrosination in *TTL^−/−^* mice, PCN neurons almost lack tyr-tubulin and mainly contain detyrosinated tubulin, called glutamylated tubulin (glu-tubulin) (supplemental Figure S1). To analyze the effect of the absence of retyrosination in neurons, we compared the positioning of PCN cell bodies and axonal projections in wt and *TTL^−/−^* mice at birth. During development, the growing axons of all PCN neurons, including those that will form the lateral reticular nucleus (LRN) as well as the inferior olivary nucleus (ION), are first attracted toward the floor plate and cross it to reach their cerebellar target. In contrast, while the cell bodies of LRN neurons cross the floor plate, those of ION neurons stop before crossing it and develop an axonal inter-olivary commissure.

**Figure 1:**
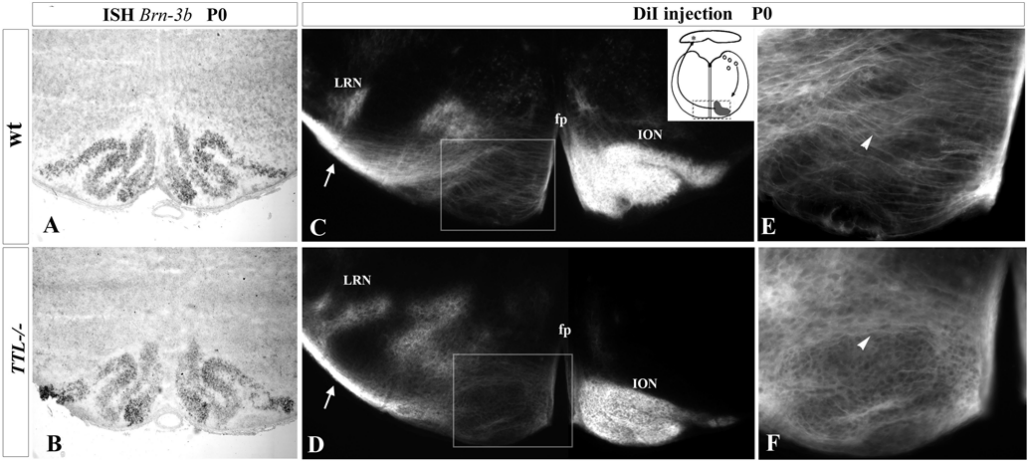
Correct positioning of ION cell bodies but impaired aspect of ION fibers in *TTL* ^−/−^ newborn mice. (A, B) At birth, a *Brn-3b* probe allowed the visualization of the whole ION (Inferior Olivary Nucleus) lamellae in wt (A) and *TTL* ^−/−^ mice (B) upon ISH. ION rostral lamellae were properly developed in *TTL*−/− mice. (C–F) Upon unilateral DiI injections in the cerebellum (left side and schema in C) and retrograde PCN labeling at birth, in wt mice (C), the ION located controlateral to the injection site and the ipsilateral lateral reticular nucleus (LRN) were labeled. Crossing fibers of the olivary commissure were visualized ventrally in the olivary region (white arrowhead in E). In *TTL^−/−^* mice, ION neurons were also labeled controlaterally to the injection site (D), but showed a disorganization of ION fibers in the ION mass, once they have crossed the floor plate (fp) and locate ipsilaterally to the injected cerebellum that they reach (white arrowhead in F). LRN was correctly labeled ipsilaterally (D). For each genotype, 4 newborn mice were analyzed after DiI injection.

To determine the position of cell bodies, we used a *Brn3b* mRNA probe to visualize ION neurons and a *Tag1* probe for neurons of the LRN. No delay was observed in the time ION neurons needed to get their proper position close to the floor plate at birth (Fig. 1A, B; n = 3 for each stage). The ION cell bodies showed their characteristic lamellated organization in both wt (Fig. 1A) and *TTL^−/−^* mice (Fig. 1B). The LRN cell bodies were also correctly located laterally to the ION in the absence of *TTL* (data not shown).

In parallel, we analyzed the PCN axon development toward their cerebellar target in vivo. In this purpose, we performed retrograde tracing of PCN axons after unilateral insertion of a crystal of DiI in the cerebellum at birth (schema in Fig. 1C). ION neurons were labeled contralaterally to the injection site, and LRN neurons ipsilaterally in both wt (n = 8; Fig. 1C) and *TTL^−/−^* mice (n = 5; Fig. 1D). However, the aspect of post-crossing olivary fibers, growing through neurons forming the other contralateral ION mass, showed some defects in *TTL* ^−/−^ mice. Instead of them being all perpendicularly oriented to the floor plate as in the wt (Fig. 1C and 1E at higher magnification), the olivary fibers seemed to navigate around the cells to leave the ION masses on the opposite side and to lose their straight orientation in *TTL^−/−^* hindbrains when located in the vicinity of the floor plate (Fig. 1D and 1F at higher magnification). The ION phenotype is the same in all cases, excluding the hypothesis of variable penetrance.

These results suggest that, in vivo, the axon pathway is impaired when axons from *TTL^−/−^* embryos navigate in the vicinity of their intermediate target, the floor plate, that is a source of both diffusible and contact cues [11] during ION axon outgrowth and neuronal migration. Thus, we decided to further investigate the responses of *TTL^−/−^* neurons in response to guidance factors using in vitro assays, focusing on axons pathfinding.

### *TTL* ^−/−^ growth cones show a hypertrophic morphology in the vicinity of the floor plate in organotypic hindbrain cultures

**Figure 2:**
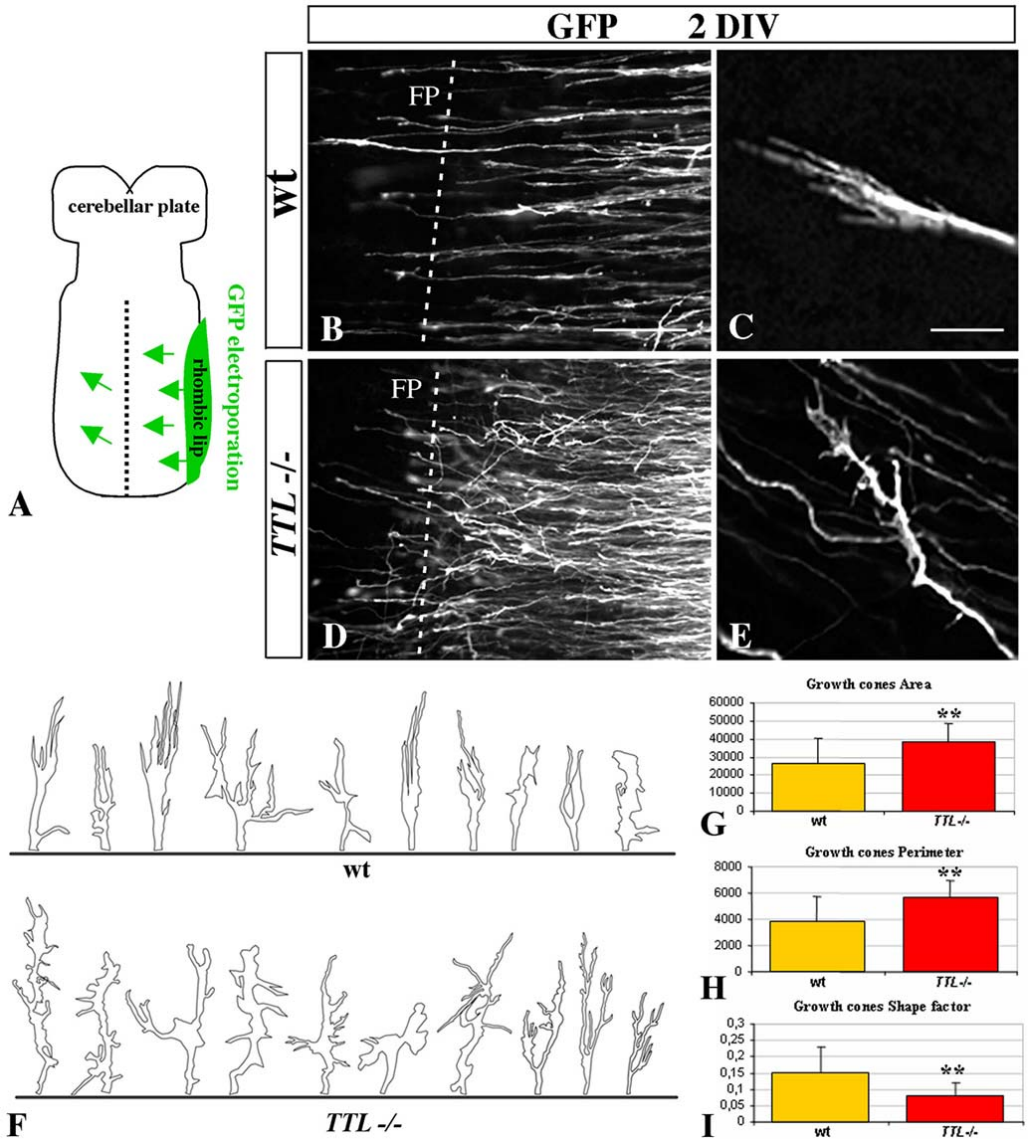
The morphology of the growth cone is impaired at the vicinity of the floor plate in *TTL^−/−^* embryos. (A) Schematic representation of hindbrain organotypic cultures from E12.5 embryos after unilateral GFP-electroporation of the rhombic lip. After 2 DIV, both wt (B) and *TTL^−/−^* (D) GFP-electroporated PCN axons have reached the floor plate (FP, dashed line) but their axonal pathway was impaired in *TTL^−/−^* embryos. In wt axons, the growth cones showed a thin aspect, with all filipodia oriented with a straight alignment toward the floor plate (B and C at higher magnification), whereas in *TTL^−/−^* embryos, they showed a more complex morphology, emitting numerous non-oriented filopodia (D and E at higher magnification). Quantification of growth cones size was performed from drawings of 20 wt and 20 *TTL* ^−/−^ GFP-electroporated neurons (F). Their area (G), perimeter (H) and shape factor (a function of both area and perimeter indicating how far from a circular shape growth cones are) (I) have been calculated. Data are presented using arbitrary units. ** *P<0,01*. Scale bars: 30 µm in B (forB, D); 5 µm in C (for C, E).

To mimic axon outgrowth in vivo and observe *TTL* ^−/−^ neurons outgrowth in their physiological environment, we developed organotypic cultures of hindbrains of E12.5 embryos, as they only navigate inside the hindbrain as a substrate. To further compare the aspect of individual axons and growth cones, a GFP-expression plasmid was electroporated unilaterally at the rhombic lip (Fig. 2A). To analyze whether the growth speed was affected by the absence of tyrosination, we fixed GFP-electroporated organotypic cultures after 30, 48 and 72 hours and confirmed that axon outgrowth could develop ex vivo with a proper time course and amplitude. After 2DIV, when they reached the vicinity of the floor plate, wt axons were all oriented parallel to each other (Fig. 2B) and showed a thin growth cone with all filopodia oriented straight toward the floor plate (Fig. 2C). On the contrary, in *TTL* ^−/−^ hindbrains, the trajectory of the axons was disorganized (Fig. 2D). In addition, at higher magnification, the growth cone showed a hypertrophic aspect, with randomly oriented filopodia (Fig. 2E and drawings in Fig. 2F), not at the initiation of migration but especially when growth cones are in the vicinity of the floor plate (supplemental Figure S2). Thus, the most obvious phenotype is observed close to the floor plate, although we cannot exclude a slighter difference all along the pathway, that would be hard to detect and evaluate through the present techniques.

To further analyze these morphological changes, we have quantified the growth cone area and perimeter in wt (n = 20) and *TTL* ^−/−^ (n = 20) neurons. Both areas (26 295 a.u. (arbitrary units) and 38 676 a.u. respectively) and perimeters values (3 825 a.u. and 5 634 a.u. respectively) were significantly increased in *TTL* ^−/−^ growth cones (*P*<0,01) (Fig. 2G and H respectively). The resulting shape factor (see Material and Methods) was 0.15 in wt and 0.08 in *TTL* ^−/−^ growth cones (*P*<0,01) (Fig. 2I), which indicates that the morphology of *TTL* ^−/−^ growth cones is more complex than the wt one. In conclusion, in these physiological conditions, there was no delay in the leading process outgrowth in *TTL* ^−/−^ hindbrains, but the morphology of growth cones appeared to be particularly affected when they navigate close to the floor plate.

### In vitro, *TTL* ^−/−^ axons that grow in presence of soluble Netrin-1 or toward Netrin-1 in a collagen matrix show a decreased outgrowth

**Figure 3:**
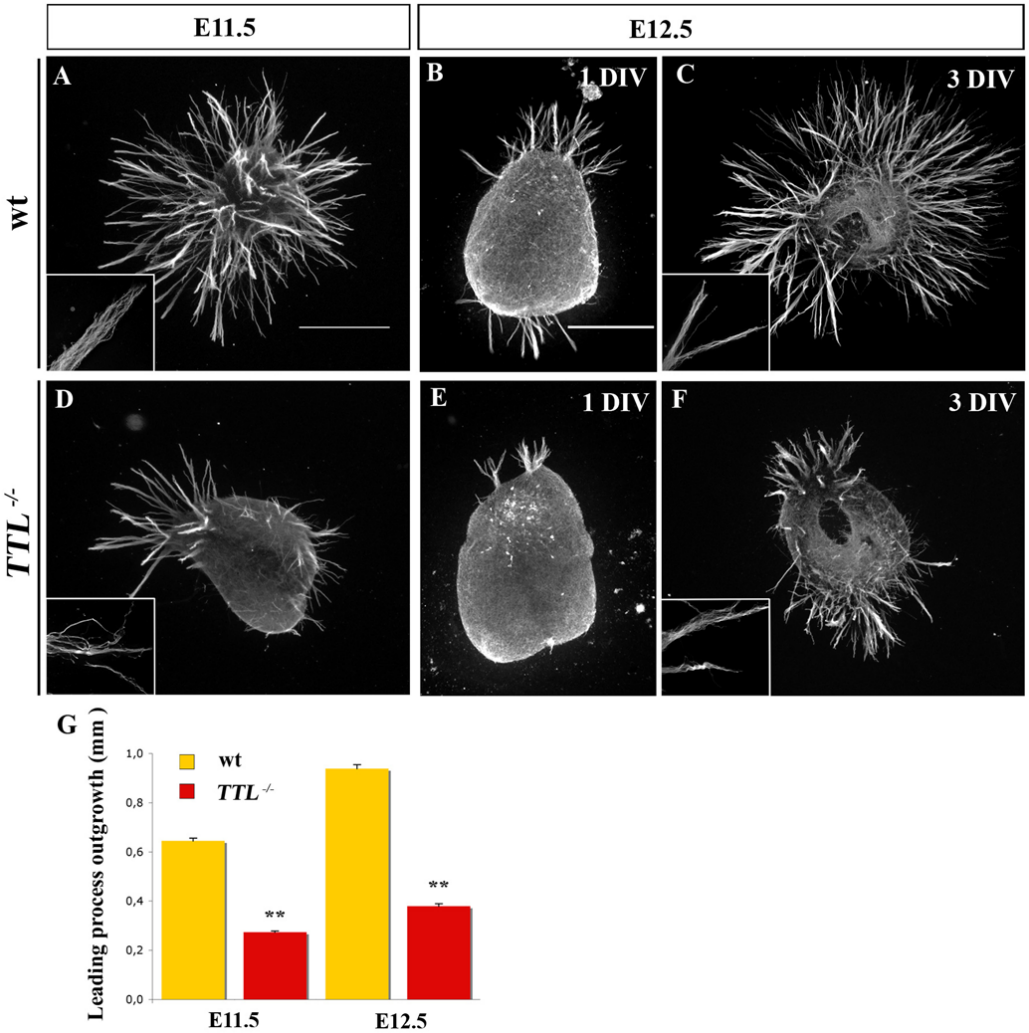
In a collagen matrix, axon outgrowth is reduced with *TTL^−/−^* precerebellar neurons. E11.5 and E12.5 explants were cultured in a medium containing soluble Netrin-1. The leading processes outgrowth was then analyzed after βIII-tubulin staining. After 3DIV, the outgrowth in wt explants were significantlymore developed in wt explants at E11.5 (n = 9) and E12.5 (n = 6) (A, C) than in *TTL^−/−^* explants (D, F). (B, E) After 1DIV only, the decreased outgrowth was already detectable. Inserts in A, C, D and F show high magnifications of fibers in fascicles that showed a disorganized aspect in the absence of TTL compared to the parralel organization of leading processes inside fascicles in wt. (G) Bar graphs showing outgrowth areas were obtained upon threshold quantification. **P<0,001. Scale bars: 500 µm in A and 300 µm in B.

First, we analyzed the possible role of tubulin retyrosination on leading process outgrowth using collagen assays. Explants of rhombic lips at E11.5 (n = 9) and E12.5 (n = 6) were first cultured with conditioned medium from Netrin-1 secreting cells in a collagen matrix. Netrin-1 was previously shown to promote PCN axon outgrowth in collagen assays and to be necessary for PCN nucleokinesis in vitro [17]. Since the leading edges of E11.5 migrating neurons is the future axon and at E12.5, leading processes have axonal growth cone-like morphology, we will refer to axons and axon-like as leading processes in quantifications at E11.5 and E12.5 respectively. When cultured with soluble Netrin-1, the quantification of the total outgrowth of the leading processes by TUJ1 immuno-staining and thresholding revealed that their growth was reduced about 3 times in *TTL^−/−^* explants, after 3 days in vitro (3 DIV) (Fig. 3A, C, D, F and G). In addition, axon-like fascicles that grew from *TTL* ^−/−^ explants showed a fuzzy organization with non parallel leading processes inside the fascicles (compare high magnifications in wt and *TTL* ^−/−^ in Fig. 3A, C, D and F). It is noteworthy that this decrease in leading processes outgrowth was already observed after 1 DIV (Fig. 3B and E).

**Figure 4:**
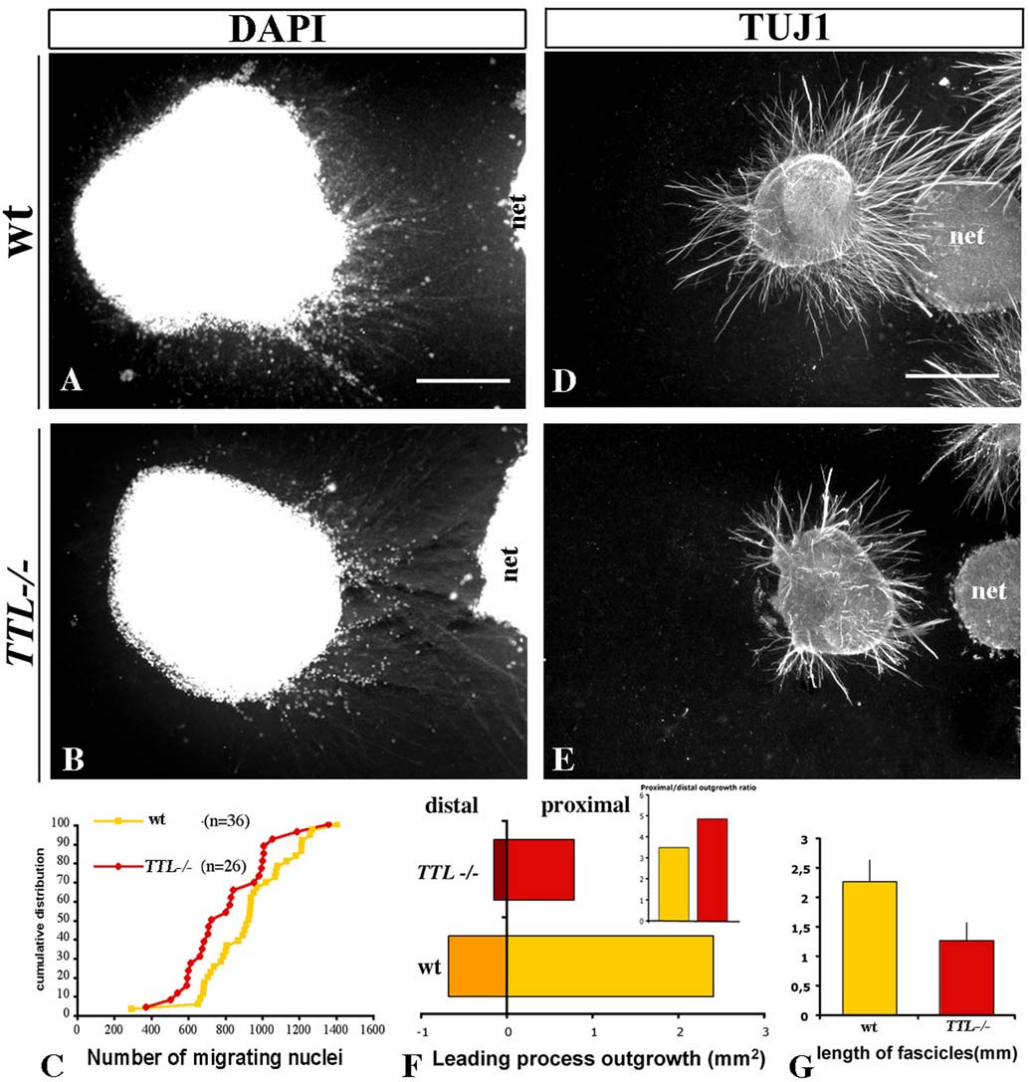
Analysis of axon outgrowth from *TTL^−/−^* explants toward a local source of Netrin-1 compared to wt explants. E12.5 rhombic lip explants containing PCN neurons were faced with Netrin-1-secreting cells (net1) and cultured for 3 days. Nucleokinesis and axon outgrowth were analyzed with DAPI and TUJ1 antibody stainings respectively. (A, B) Nuclear migration from wt and *TTL* ^−/−^ explants toward Netrin-1 secreting cells did not show any difference. (C) Cumulative distribution quantification of nucleokinesis from wt and *TTL* ^−/−^ explants. (D, E) Axon outgrowth in wt explants (D) was more developed than in *TTL^−/−^* explants (E). (F) Bar graphs showing total axon outgrowth toward (proximal) and away (distal) from the Netrin-1 source were obtained upon threshold quantifications. The proximal versus distal ratio outgrowth was not altered in *TTL* ^−/−^ explants, but the total axon outgrowth was reduced about 3 times. (G) Bar graph showing quantification of the length of axon fascicles growing out of the rhombic lips explants measured for 20 fascicles of each explant from wt and *TTL* ^−/−^ embryos. n = 36 explants for wt and n = 26 for *TTL* ^−/−^ for every quantification. * *P<0,05*. Scale bars: 250 µm in A (for A, B), 500 µm in D (for D, E).

Since Netrin-1 is secreted by the floor plate and because in vivo and ex vivo defects were observed close to the midline, we tried to determine whether this alteration was due to a defect in the ability of *TTL^−/−^* neurons to transduce the Netrin-1 attractive effect. For that purpose, we faced rhombic lip explants with a local source of Netrin-1. The rate of nucleokinesis was not significantly affected in the *TTL* ^−/−^ explants when compared to the wt ones as illustrated for E12.5 neurons (Fig. 4A, B, and C). Furthermore, the outgrowth was similarly reduced in this condition but leading processes still grew preferentially toward the Netrin-1 source in the proximal quadrant rather than in the distal quadrant (Fig. 4D, E, and F), indicating that *TTL* ^−/−^ neurons can still respond to guidance cues. These observations were consistant with their globally normal oriented outgrowth in and ex vivo and the prediction that there is no defect in guidance. Nevertheless, we observed that leading processes from *TTL* ^−/−^ neurons were thicker and shorter as revealed by individual length measurements (Fig. 3D and F and Fig. 4E and G) than those growing from wt explants (Fig. 3A, C and Fig. 4D).

To get a better understanding of this decreased outgrowth not previously detected in and ex vivo, we further analyzed the behaviour of PCN leading processes during pathfinding toward Netrin-1.

### Neurites from *TTL* ^−/−^ PCN neurons branch extensively and grow through tortuous trajectories toward Netrin-1

**Figure 5:**
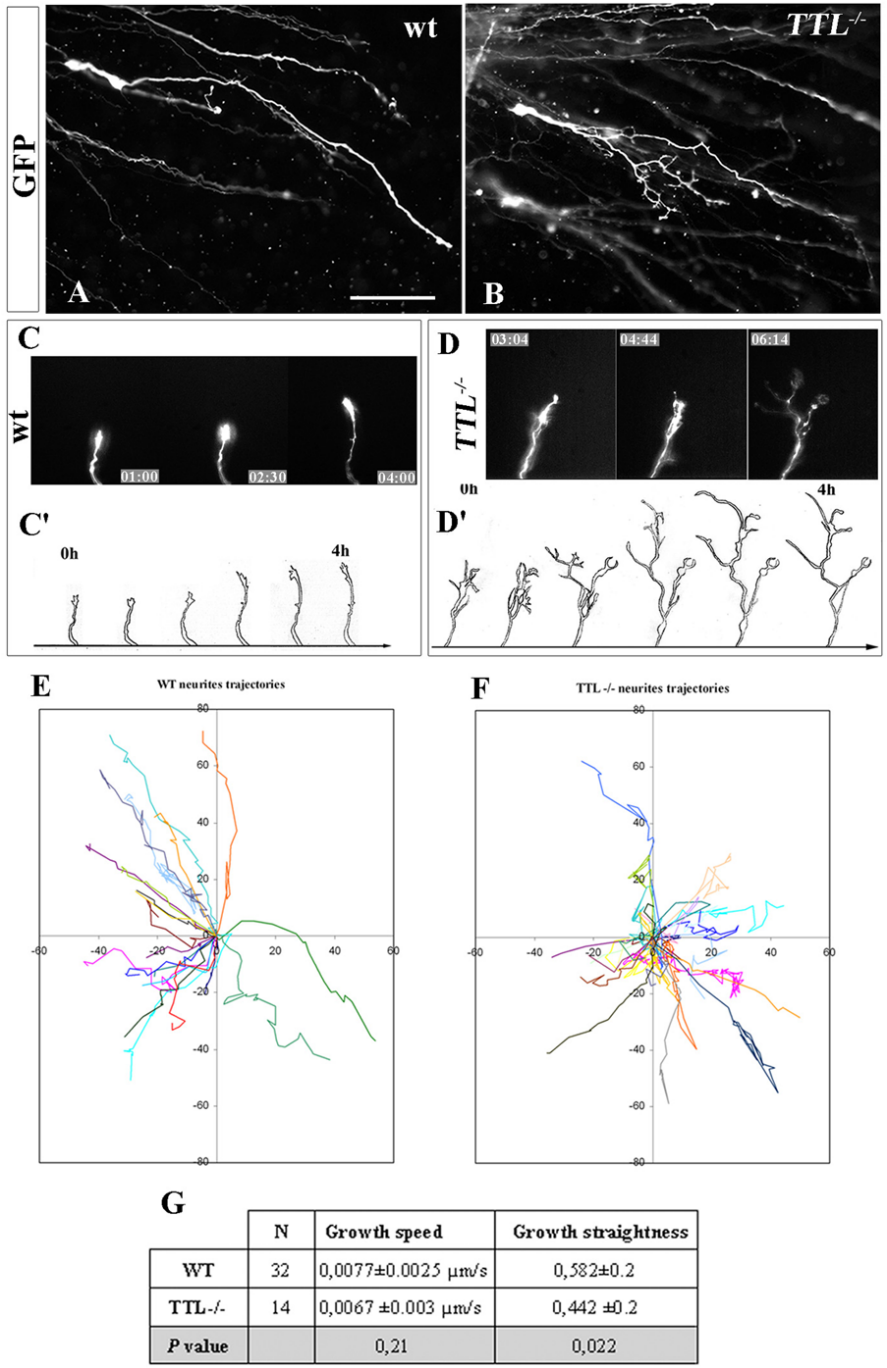
Extensive branching and tortuous trajectories of neurite tips of *TTL* ^−/−^ neurons in the collagen matrix. (A, B) Single morphology of migrating PCN neurons from rhombic lips explants after GFP electroporation showed that wt neurons grew straight, without emitting secondary neurites (A) while *TTL* ^−/−^ neurons often emitted numerous secondary neurites (B). (C, D) The behaviour of growth cones at the distal tip of single GFP-electroporated wt (C and Movie S1 in supplementary material) and *TTL* ^−/−^ neurons (D and Movie S2 in supplementary material) growing in the collagen matrix was dynamically analyzed by time-lapse experiments after 2 DIV. While wt neurons grew straight toward the Netrin-1 source without any branching, numerous branches were observed in the distal part of *TTL* ^−/−^ growing axons. C' and D' are drawings of 4 hours lasting sequences from Movie S1 and Movie S2, illustrating the morphological differences between wt and *TTL* ^−/−^ axon distal tip respectively. (E, F) Trajectories of distinct neurites tips from wt (E) or *TTL* ^−/−^ (F) GFP-electroporated neurons recorded in the collagen matrix. Most wt neurites show a very straight trajectory (E), contrary to *TTL* ^−/−^ neurites that often exhibit more complex behaviours, with tortuous trajectories and forward and backward movements (F). (G) The growth speed and growth straightness of wt and *TTL* ^−/−^ neurites have been quantified and show that *TTL* ^−/−^ neurites are able to grow as fast as wt ones but their trajectories are less straight, indicating a dispersion of their pathway. Scale bar: 30 µm in A (for A, B).

**Figure 6:**
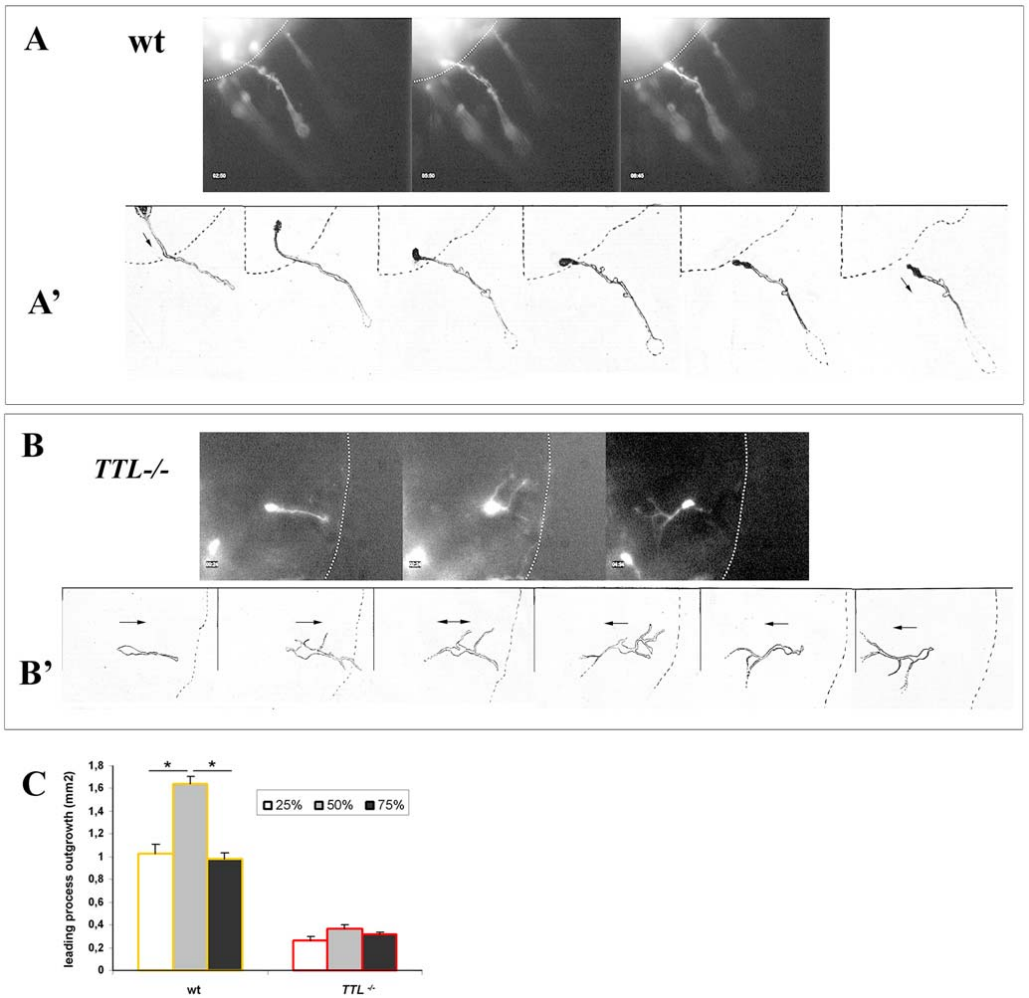
Distinct behaviours of E12.5 GFP-electroporated PCN neurons from wt and *TTL* ^−/−^ embryos at the edge of rhombic lip explants. (A, B) Single GFP-electroporated neurons from wt (A and Movie S3 in supplementary material) and *TTL^−/−^* embryos (B and Movie S4 in supplementary material) were recorded after 2 DIV, inside the explants, at the moment they intended to leave it to enter the collagen matrix to go toward the Netrin-1 source. While wt neurons could easily exit the explants to enter the collagen matrix, *TTL^−/−^* neurons emitted numerous primary or secondary sensing neurites when contacting the edge of the explants (dashed line) and failed to enter the collagen matrix, finally changing their growth direction. Drawings of single neurons provided a sequential representation of the behaviour of wt (A') and *TTL^−/−^* (B') neurons. Black arrows in drawings indicate the direction of migration. (C) Bar graphs showing quantification of total axon outgrowth of wt and *TTL^−/−^* explants placed in collagen matrix of distinct concentration. Changing the density, and thus, the softness of the substrate, has no effect on total axon outgrowth of *TTL^−/−^* neurons.

After electroporation of GFP in the hindbrain and subsequent collagen assays of PCN neurons, the morphology of individual leading processes in both wt and *TTL* ^−/−^ neurons could be visualized. As previously reported [18], in wt PCN neurons, leading processes grew straight toward Netrin-1, and branching was never observed (Fig. 5A). In contrast in *TTL* ^−/−^ PCN neurons, secondary neurites developed extensively (Fig. 5B, Supplemental Figure S3, also illustrated in Fig 6B).

The pathfinding of the leading processes was recorded in collagen matrix when axons were located out of the rhombic lip explant. We have focused on growth cone movements after GFP electroporation. *TTL* ^−/−^ axons were able to grow among other fasciculated axons but either retracted or stopped growing further when they reached the tip of the fascicles. We have observed that the distal part of axons showed a more complex morphology in *TTL* ^−/−^ neurons due to extensive branching compared to wt ones, that never branched (see wt and *TTL^−/−^* Movie S1 and Movie S2 respectively in supplementary material, and Fig. 5C and D). Drawings of the axonal tips development along 4 hours illustrate the presence of numerous neurites in the distal part of *TTL* ^−/−^ axons (Fig. 5D') compared with wt (Fig. 5C').

Tracking of neurite trajectories revealed that most wt neurons showed a straight trajectory of their neurite tips, contrary to *TTL^−/−^* neurons that exhibited a more complex behaviour, with tortuous trajectories and forward and backward movements (compare Fig. 5E and F). Quantification of the mean deviation from the straight line provided a growth straightness factor, which was significantly higher in wt than in *TTL^−/−^* neurites (Fig. 5G), which confirms the dispersion of their trajectories. Despite these complex axonal pathways and growth cones morphologies, the axonal growth speed was not affected in *TTL* ^−/−^ neurons compared with wt (Fig. 5G). Altogether, these observations of the axonal tips behaviours could at least partly explain the decreased length of axon fascicles observed when axons grew toward Netrin-1 in collagen assays (Fig. 4).

### Neurons with detyrosinated microtubules fail to enter the collagen matrix

In parallel, we have also analyzed axon pathfinding inside rhombic lips explants by time-lapse experiments since it seemed that most of the GFP-electroporated neurons remained inside the explant from *TTL^−/−^* embryos. Surprisingly, wt and *TTL^−/−^* PCN neurons showed distinct behaviours when leaving the explant to enter the collagen matrix. Actually, inside the explants, the wt GFP-electroporated neurons emitted a single primary neurite that entered the collagen matrix straightaway toward the Netrin-1 source (see Fig. 6A and Movie S3 in supplementary material). Conversely, the aspect of GFP-electroporated *TTL^−/−^* neurites was often modified when they contacted the edge of the explants as they started to emit an increased number of primary and secondary neurites. By this way, they seemed to explore extensively the environment and the border of the explant, but few of them succeeded in getting out of the explant to enter the collagen matrix (see Fig. 6B and Movie S4 in supplementary material), which could explain the decreased total axon outgrowth upon TUJ1 staining and threshold quantification, observed from 1 DIV (Fig. 3 and Fig. 4). Drawings from 4 hours movies of wt and *TTL* ^−/−^ neurons at the edge of the explant allowing a better representation of their respective behaviours are shown in Figures 6A' and B'.

Thus, we conclude that *TTL* ^−/−^ neurons are still able to emit leading processes and to detect and transduce guidance signals, but their ability to leave the explant and enter the collagen matrix is decreased. Actually, this may reflect a difficulty for leading processes with detyrosinated MTs to move forward through a soft tridimensional substrate like collagen, that may offer less adhesion sites for growth cones than their physiological environment. Although it is admitted that the behavior of some cells can vary on substrates of various stiffness, very few molecular cues have been proposed ([19] for a review). We have tested the role of stiffness/softness for PCN outgrowth and their link with MTs tyrosination by culturing rhombic lip explants in collagen matrices of increasing concentrations, 25%, 50% and 75% (Fig. 6C). Interestingly, in wt explants, the maximum outgrowth was reached with an intermediate concentration of collagen (50%), whereas when the collagen was diluted (25%) or concentrated (75%), the total outgrowth of the leading processes decreased to similar levels (Fig. 6C). These results indicate that the density of the matrix can indeed have significative effects on leading process outgrowth in wt neurons. It suggests that an intermediate softness could be required to get the formation of enough adhesion sites without impeding the normal progression of the growth cone through the matrix, because of its density. At the reverse, collagen density has less effect on *TTL* ^−/−^ explants since we did not succeed in increasing their outgrowth neither by increasing nor by decreasing the collagen concentration (Fig. 6C), pointing out that collagen seems to be a more challenging type of substrate for *TTL* ^−/−^ growth cones than for wt ones, but its relative softness does not seem to affect the outgrowth of *TTL* ^−/−^leading processes.

Altogether, these in vitro experiments in collagen matrix showed that *TTL^−/−^* leading processes have no guidance defect which was consistant with our in vivo observations. However, they brought out a defect that remained subtle in vivo only visible through the morphological leading processes disorganizations around the floor plate, which is the abnormal extension in certain substrata.

### The cytoskeletal organization of the growth cone is impaired in *TTL^−/−^* neurons

To further analyze intracellular changes that could be involved in morphological changes of the growth cone and pathfinding modifications in detyrosinated MTs, we have analyzed the respective localization of F-actin and MT filaments in wt growth cones or when tyr-tubulin was substituted by glu-tubulin at the extremity of the *TTL* ^−/−^ axons.

**Figure 7:**
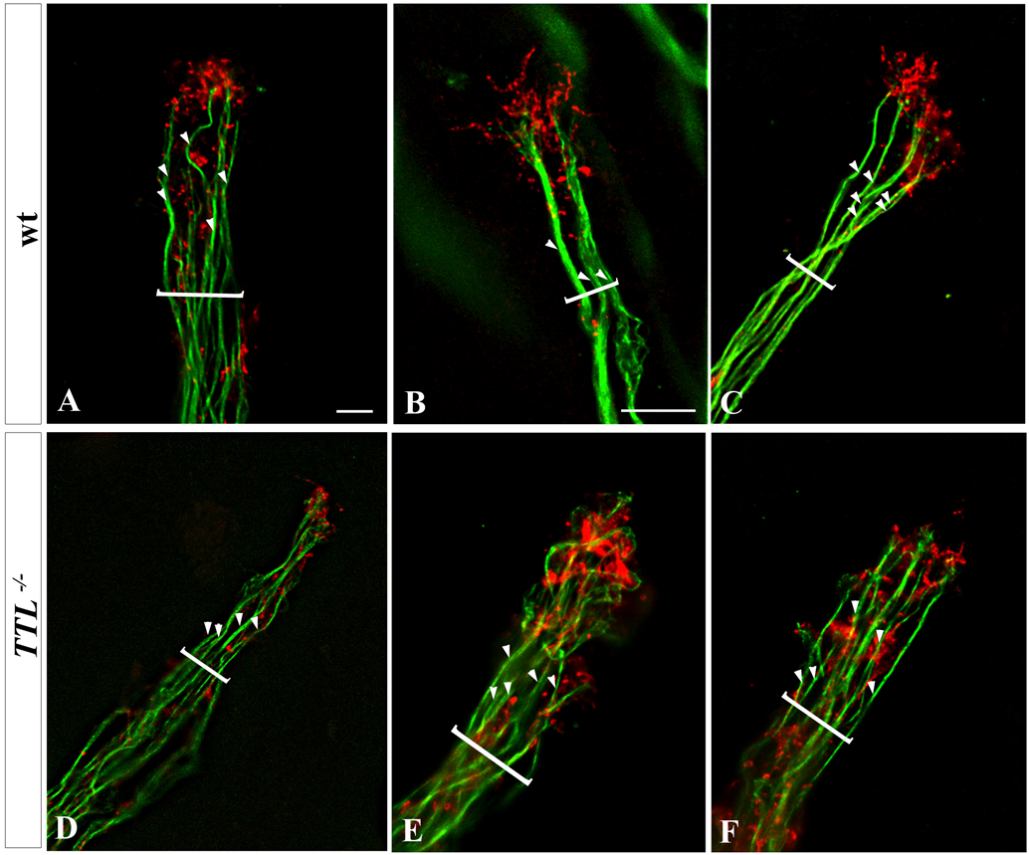
Impaired Actin-MT cytoskeleton relative organization in growth cones of *TTL* ^−/−^ neurons. Picture acquisitions of the distal part of several bundled E12.5 PCN axons after 3 DIV in collagen matrix labelled with an anti-total α-tubulin antibody (green) and phalloidin (red) from wt (A, B, C) and *TTL* ^−/−^ neurons (D, E, F) were deconvoluted (left pannels). Brackets indicate axons bundles and white arrowheads points single axons. In wt, the F-actin present in the peripheral domain of growth cones formed a cap at the distal tip of axons bundles, around the tubulin labeling, whereas in *TTL* ^−/−^ neurons, MTs entered more deeply in the F-actin domain and tubulin staining overlapped with the F-actin distal cap. Scale bars: 4 µm.

We have performed double staining for F-actin and total α-tubulin in PCN growth cones in a collagen matrix and deconvolution after picture acquisition. In neurons from wt embryos, F-actin was located distally in the growth cone [2] whereas the MT bundles were located more centrally, with only some isolated MTs contacting and barely entering the F-actin domain (Fig. 7A). Thus, few overlapping between F-actin and microtubules could be observed and a large peripheral domain containing F-actin only was observed distally. Conversely, in *TTL^−/−^* neurons, instead of being juxtaposed, both actin and MTs domains more widely overlapped (Fig. 7B).

This result suggested that tubulin tyrosination is required for the regulation of the global cytoskeletal organization of the growth cone.

### Intracellular cues possibly involved in defects of growth cone morphology and cytoskeleton

**Figure 8:**
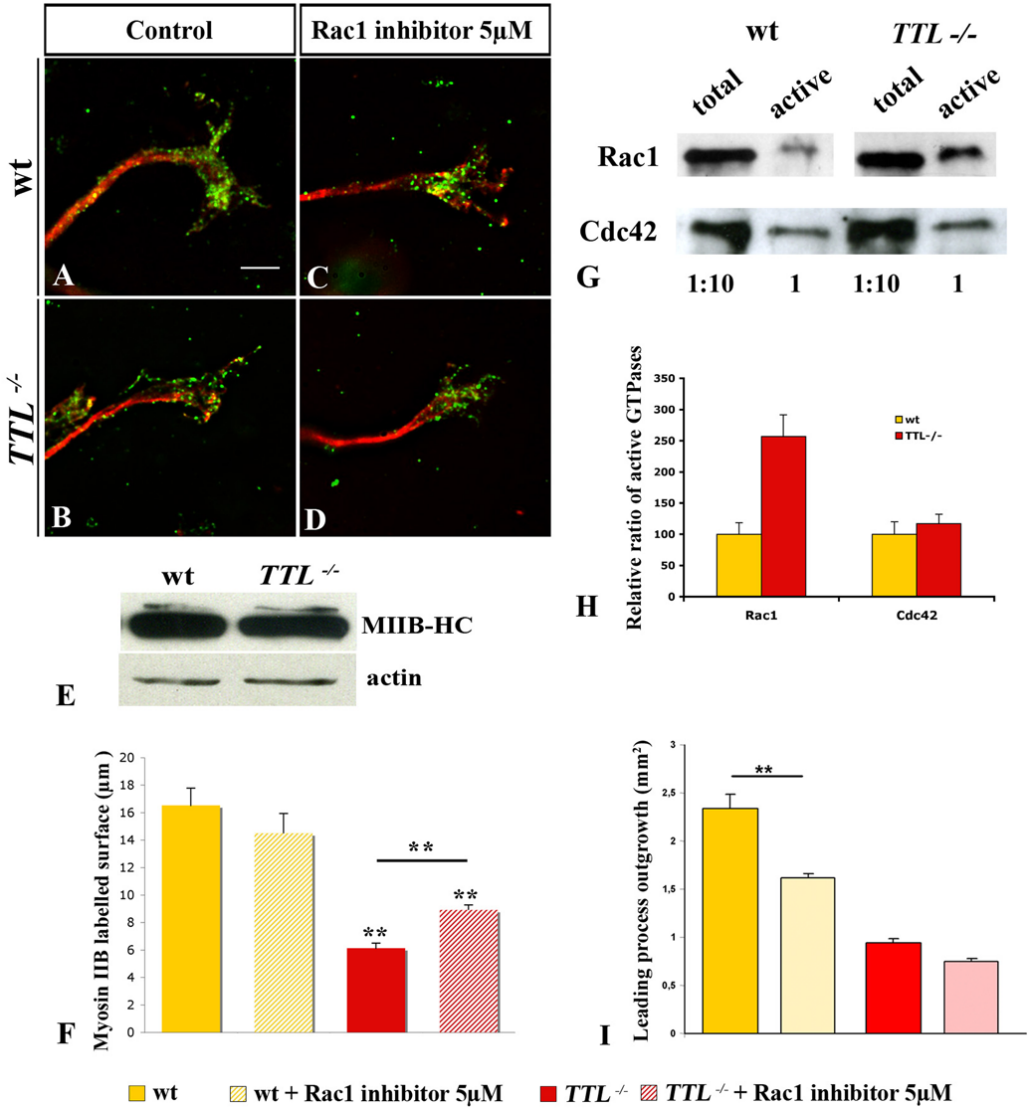
Impaired Myosin IIB recruitment at the growth cone of *TTL^−/−^* neurons. (A–D) Growth cones from E12.5 PCN neurons in collagen assays after 2 DIV. (A, B) Illustrations of double immuno-labeling of Myosin IIB heavy chain (MIIB-HC) (in green) with total α-tubulin (in red) in wt (A) and *TTL^−/−^* (B). MIIB-HC showed a punctuated staining, accumulated in wt growth cones (A), while in *TTL^−/−^* neurons, MIIB-HC distal recruitment was decreased in the growth cone, or even absent is some cases (B). (C, D) When cultured in the presence of 5 µM of Rac1 inhibitor, MIIB-HC still accumulated distally in wt. In contrast, in *TTL^−/−^* neurons, a partial distal recruitement is recovered after treatment with 5 µM of Rac1 inhibitor. (E) The expression level of MIIB-HC in wt and *TTL^−/−^* hindbrains extracts from wt and *TTL^−/−^* E12.5 embryos was analyzed by Western blot revealed with the anti-MIIB-HC antibody. The expression level of MIIB-HC was not affected in the absence of tubulin retyrosination in *TTL^−/−^* neurons. (F) Bar graphs show MIIB-HC labeling quantification using threshold technique and Metamorph analysis. No significant difference could be detected in wt explants cultured in control conditions (n = 31) and drug treated (n = 20). In *TTL^−/−^* growth cones, MIIB-HC is significantly mis-localized (n = 40). The distal part of *TTL^−/−^* axons cultured with Rac1 inhibitor show a partial but significative rescue of MIIB-HC recruitment (n = 45). (G) Quantification of Rac1 and Cdc42 GTPases activity in E12.5 wt and *TTL^−/−^* hindbrains. (H) Bar graphs show the quantification of the relative active state of each GTPase upon Western blotting quantification in both wt (yellow) and *TTL^−/−^* (red) lysates. The ratio of active GTPases in *TTL^−/−^* versus wt hindbrains showed an increase of Rac1 activation whereas the ratio of Cdc42 activation remained unchanged when tubulin retyrosination was defective. (I) Bar graphs show quantification of axon outgrowth from wt and *TTL^−/−^* explants with or without 5 µM of Rac1 inhibitor. ** *P*<0,001. Scale bar: 2 µm.

Myosin IIB is one of the main components of the growth cone that is required in growth cone motility through the regulation of actin retrograde flow and MTs retrograde movement [20], [21], [22], [23]. Thus, we have investigated Myosin IIB localization in *TTL^−/−^* neurons through Myosin IIB heavy chain (MIIB-HC) immuno-staining in collagen assays (Fig. 8A, B). We have quantified MIIB-HC labeling in the growth cone by thresholding the immuno-labeled aera and quantification with Metamorph, both in wt and *TTL^−/−^* growth cones (Fig. 8F). MIIB-HC accumulated in growth cones of wt PCN neurons (Fig. 8A). Conversely, in *TTL* ^−/−^ neurons, MIIB-HC accumulation in growth cones was significantly decreased (Fig. 8B). Note that these values might be underestimated as TTL−/− growth cones are larger as compared to wt. In addition, we have checked that the total amount of MIIB-HC in *TTL^−/−^* hindbrains is not changed by western blot analysis (Fig. 8E). Thus, it seems that the tyrosination on MTs +TIPS is required for the local recruitment of Myosin IIB in the growth cone. Myosin IIB light chain, active and total, was also still expressed and detectable in *TTL* ^−/−^ mutant axons in PCN primary dissociated cultures, but no more distally accumulated as observed in wt. Unfortunately, in collagen matrix, the poor quality of the antibody or the very low expression of the light chain did not allow its detection.

Others well known regulators of both microtubules and actin cytoskeleton are the small GTPases [24], that, especially Rac and Cdc42, control the growth cone morphology and the formation of filopodia and lamellipodia depend on the regulation of actin filaments [25]. Growing evidence suggest that Rho GTPases can regulate both MT and actin cytoskeletons that, in turn, can also directly or indirectly regulate Rho GTPases activation [26], [27], [28], [29], [30]. Thus, we have investigated the activation state of small Rho GTPases, as small Rho GTPases normally cycle between a GDP-bound inactive state and a GTP-bound active state. For this purpose, extracts were prepared from hindbrains of E12.5 wt or *TTL* ^−/−^ embryos and processed for GST-pull down. We had previously shown that, in the hindbrain, most of active small GTPases are located in PCN and in the facial motor nucleus. Thus, the use of whole hindbrains, required to get enough material from individual KO in a littermate, allows the analysis of their activity quite specifically in these neuronal nuclei [31]. The ratio of active Rac1 was increased by 3.35 times in neurons from *TTL^−/−^* embryos compared to wt embryos while the ratio of active Cdc42 remained unchanged in presence or absence of tyrosinated MTs (Fig. 8G and H).

Thus, the increased activity of Rac1, which can regulate actin filaments organization in neuronal growth cones, could be partly responsible for the changes in the growth cone morphology and cytoskeleton organization and for the impaired regulation of neurites extension revealed by the increased number of neurites all along the axons of *TTL^−/−^* neurons.

To further investigate how tubulin tyrosination could be related to the subcellular localization of MyosinIIB, we have tested the effect of inhibiting Rac1 over-activation by applying a commercial Rac1 inhibitor in collagen assays (Fig. 8C and D). We have established a dose response curve and chosen the concentration that would allow the minimal defects in outgrowth and no defect in nucleokinesis on wt neurons that is 5 µM Rac1 inhibitor (supplemental Figure S4). When applied on explants in collagen assays, we observed that the outgrowth was decreased by 30% for wt neurons and only by 20% in *TTL^−/−^* neurons (Fig. 8I). This suggested that the sensitivity of mutant axons, that carry a Rac1 over activation, was lower than the one of wt. In *TTL^−/−^* growth cones, upon application of 5 µM Rac1 inhibitor, myosin IIB distal recruitement was rescued (compare Fig. 8B and D). After quantification with or without drug application, we observed a partial and significant recovery of the distal accumulation of MyosinIIB upon down-regulation of Rac1 over-activation (Fig. 8F). Thus, Rac1 over-activation was, at least in part, responsible for the absence of Myosin IIB in *TTL^−/−^* growth cones, suggesting that it could exist a direct link between myosin delocalization in the growth cone and Rac1 over-activation. Indeed, in N1E-115 cells, it has been shown that over-expression of a constitutively active form of Rac induces cell spreading accompanied by a loss of cortical Myosin II heavy chain [32]. Here, Myosin distal recruitment is dependent on the proper regulation of Rac1 activity and occurs downstream of Rac1.

## Discussion

In this report, we show that tubulin retyrosination plays a major role in the morphology and functionality of the growth cone. We demonstrate that axons in which tubulin retyrosination does not occur emit supernumerary neurites ex vivo and in vitro. In vitro assays reveal a reduction of axon length and their axon trajectories are not straight when they grow in a collagen matrix, or ex vivo when they approach the floor plate. The pathway defects reported here in *TTL* ^−/−^ mutant hindbrains are specially emphasized around the floor plate, possibly due to its composition in both adhesion and diffusible guidance molecules, but they could also reflect a general growth defect or sensitivity in the absence of TTL. In addition, the absence of tubulin retyrosination prevents the proper intracellular organization of the growth cone cytoskeletal components. Nevertheless, *TTL* ^−/−^ axons can still follow their global pathway in/ex vivo and reach their target. In the light of our results, we will discuss the alternative growth strategies used by axons when their main sensor, i.e the growth cone, is not properly formed as in *TTL* ^−/−^ neurons. We will also discuss which intracellular associated components could link tubulin tyrosination to the phenotypes of the *TTL* ^−/−^ PCN neurons.

### Tubulin tyrosination is a major cue for growth cone morphology

The growth cone motility depends on the one hand on its function of detector and transducer of extrinsic guidance cues and on the other hand, on the traction forces it generates. MT assembly at the growth cone is important for the proper organization of the neurite cytoskeleton in growing neurites. Some of the factors that may influence MT assembly in growth cones may be linked to post-translational modifications of tubulin itself [33]. We report here that, in vitro, PCN neurons with only detyrosinated MTs emit secondary distal neurites in a repeated and uncontrolled way. Their growth cones show numerous non-oriented filopodia which confer them a complex morphology. Interestingly, other mutant mice carrying mutations in microtubule associated proteins, in particular Kif2A MT-based motor protein and Microtubule Associated Protein 1B (MAP1B), show an increased growth cone surface in vitro and an increased branching in various neuronal cell types [34], [35]. The extensive branching reported in *kif2a^−/−^* and *Map1b^−/−^* neurons was suggested to be partly due to an impaired suppression of collateral branches extension [34], [36]. In both *Map1b^−/−^* and *kif2a^−/−^* mutant mice, axon branching is also associated with guidance defects of the migratory process, that lead to impaired formation of PCN nuclei. Neurons lacking MAP1B have a reduced proportion of tyrosinated MTs and recently, it has been shown that MAP1B protein interacts with TTL [37]. Despite similar axon branching and growth cone morphology defects, PCN migration and axon responses to guidance factors seem to occur properly in *TTL^−/−^* mutant mice, which shows that all these events are not strictly linked and may be finely and locally modulated by a post-translation modification like tyrosination.

### In vivo, axons could set up compensatory mechanisms to ensure axon outgrowth

Remarkably, in vivo and ex-vivo, the *TTL^−/−^* phenotype is less dramatic than in collagen assays, since axon outgrowth is not impaired, but only the morphology of the growth cone at decision points like the floor plate is abnormal. The phenotypic difference of growing neurons in vitro versus ex vivo suggests that either neurons can set up compensatory mechanisms when growing in a physiological environment or in vivo, physical properties are less challenging for outgrowth than in vitro. This could allow axons to avoid the extensive emission of secondary branches and/or favor the pruning of supernumerary filopodia in response to guidance cues as observed during development when axons retract in response to repellent-cues like Semaphorin 3A that coordinates the activation of Myosin II [38].

Thus, we propose that in vitro, the successive emission and elimination of supernumerary neurites could allow axons to explore the environment, when the growth cone dynamic is impaired in a surrounding environment that cannot provide compensatory physiological cues. A previous analysis of *TTL* ^−/−^ mice revealed that the cortico-thalamic loop was not properly formed [8]. It remains to be established whether the role of tyrosinated MTs is identical in all types of axons that have to take decisions during pathfinding processes, independently of the morphology of the various migrating neurons and of the environment encountered during their development.

### Tyrosinated tubulin regulates the distribution and polarized organization of the components of the growth cone

During normal development, dynamic MTs can transiently enter the growth cone periphery. They actively explore the lamellipodia and even penetrate into the filopodia to integrate guidance signals [4]. In addition, Myosin IIB accumulates in the transition zone between the P- and C-domains [23]. In the present report, we demonstrate that in the absence of tubulin post-translational modification through the tyrosination cycle, detyrosinated MTs not only transiently explore the axonal tips, but invade the distal actin-rich domain of the growth cone. This defect could be due to the absence of Myosin IIB in the growth cone of *TTL* ^−/−^ neurons since recent data have shown that MTs can be retrogradely transported out of the P-domain to the C-domain through linkage to the retrograde actin flow, which depends on the activity of Myosin II [39]. These data suggest that MTs invasion of the P-domain of *TTL* ^−/−^ growth cones could result from defective acto-myosin contractility at the transition zone. Tyrosination could be a cue for regulating actin/MTs cross-talk, allowing Myosin IIB activation in the growth cone.

### Possible cross-regulations between tyrosinated tubulin and actin regulators

Mis-regulations of Rho GTPases activities can account for changes in neuronal morphologies. In addition, increasing data show that the cross-talk between MTs and actin is often ensured by small GTPases and their regulators. MTs and small GTPases can regulate each other, and small GTPases, then, can coordonate the regulation of both actin and MT during the migratory process in particular [27], [40]. The state of MT polymerisation or depolymerisation can affect the activation of distinct RhoGTPases [41], [42] and the activation of Rac1 is necessary for getting a MT dependent accumulation of actin filaments at the periphery of the growth cones [29]. We report that the rate of activation of Rac1 was increased, which also reflects a mis-regulation of small GTPases activity in the absence of tubulin tyrosination. Interestingly, the Rac1/Cdc42 effector IQGAP can interact with Clip-170 and mediates a transient capture and stabilization of MTs, leading to establishment of polarized cell morphology and directional cell migration [43], [44]. As reported by Peris et al. [9], Clip-170 recruitment at the plus ends of MTs is impaired in *TTL* ^−/−^ mice and this could prevent the local recruitment of IQGAP. Several other Rho GTPase regulators have been proposed to interact with +TIPS-MTs and/or actin, such as GEF-H1 [45] and Asef, a Rac1 GEF linked to MTs +TIPS through its interaction with APC [46], which phenotype in KO mice would be interesting to compare with *TTL* ^−/−^ mice. In growth cones, other actin regulators, such as Ena/VASP proteins, are concentrated at filopodia tips and control filopodial dynamics in neurons. Thus, these actin regulators also control the growth cone morphology [47]. It will be of interest to analyze Ena/VASP localization in *TTL* ^−/−^ mouse embryos to determine whether mis-localization of actin regulators can occur as a consequence of abnormal tubulin modifications. An abnormal recruitment of actin regulators or Rho GTPase regulators or effectors in the growth cone through their interaction with plus-ends tyrosinated MTs could explain the increased activity of Rac1 in *TTL* ^−/−^ embryos.

### Tyrosinated MTs could be required for growth cones to progress through unfavorable substrates

During its pathfinding, the growth cone has to ensure the generation of traction force in the substratum, required for forward advancement of the growing axon [48].

In *TTL^−/−^* neurons, axon outgrowth is decreased in collagen assays, which possibly results from the difficulties encountered by growth cones when contacting the collagen matrix at the edge of the rhombic lip explant and later on, when navigating in the collagen matrix. In organotypic culture, growth cone morphology is also affected when axons have to enter the floor plate, that provides a glial substrate. Finally, the aspect of *TTL^−/−^* olivary fibers growing through the contralateral ION is also affected in vivo. *TTL^−/−^* axons do not form the inter-olivary commissure with straight oriented axons observed in wt. Interestingly, none of the defects observed in *TTL^−/−^* axons could be detected when neurons were cultured on a two-dimensional substrate of poly-ornithine/laminin (not illustrated), which strengthens the link between the state of MTs tyrosination and the ability of an axon to grow in complex 3D substrates. Altogether, in vitro experiments with Netrin-1 in collagen matrix confirm the in/ex vivo observations: the orientation of axon pathway was not affected in *TTL^−/−^* neurons. They also brought out that, when growing in certain substrata, leading process outgrowth was decreased. The tension generated by *TTL^−/−^* growth cones may not be sufficient for their proper pathfinding through complex substrates, especially when they have to leave a favourable substrate or enter a new substrate.

Substrate adhesion depends on the linkage of the actin cytoskeleton to integrins at sites of adhesion complexes. In the growth cone, their formation and stabilization are controlled by the coordinated activities of Rac1 and RhoA GTPases and they regulate neurite outgrowth [49]. The turnover of adhesion complexes is controlled by acto-myosin contractility [50]. MTs by themeselves can modulate the spatial localization of adhesion complexes [51], having the ability to locally suppress acto-myosin contractility [52]. When the dynamic of MTs is decreased after α-tubulin deacetylation inhibition, the turnover of cell adhesions slows down [53]. In the absence of MTs retyrosination, in *TTL^−/−^* neurons, the over-activation of Rac1 and the defective recruitment of Myosin IIB in the growth cone could lead to a decreased ability to progress through some substrates, possibly due to defects in the formation of adhesion complexes.

## Materials and Methods

### Embryo processing

*TTL* heterozygous mice were generated as described in Erck *et al.* 2005 [8]. Mouse embryos were obtained from timed mating of *TTL* heterozygous mice or outbred Swiss mice (Janvier, Le Genest St Isle, France). For all culture assays, the hindbrains from E11.5 and E12.5 embryos were dissected in GBSS (Sigma), supplemented with 0,5% glucose. The treatment of all the animals used within the present research was in accordance with our institutional or national guidelines for the use of animals in scientific research.

### In situ hybridization

For in situ hybridization (ISH), mouse embryos were fixed in 4% paraformaldehyde (PF) in phosphate buffer (PB) PH 7,4 for 2 hours at room temperature. The neural tubes were dissected out, cryoprotected overnight with a 10% sucrose solution in PB and embedded in 7,5% gelatin/10% sucrose. They were frozen and serially sectioned in the frontal plane using a cryostat. ISH was then carried out on cryosections according to Causeret et al., 2004 [18]. *Brn-3.b* and *Tag1* probes were used and have been described previously [12], [54].

### DiI tracing: retrograde labeling of precerebellar neurons

After intracardiac perfusion with 1% PF and 1% glutaraldehyde in PBS, a small occipital craniotomy was performed to expose the cerebellum of newborn wt or *TTL* ^−/−^ hypomorphic mutant mice. 1,1′-Dioctadecyl-3,3,3′,3′-tetramethylindocarbocyanine (DiI carbocyanine, D282; Molecular Probes, Eugene, OR) attached to the tip of a broken glass pipette was applied, under a dissecting microscope, on one of both hemicerebella. Several small DiI crystals were inserted into the cerebellar tissue, medially and laterally to label most of the neurons projecting to this center. The brains of the injected micewere stored in 1% PF at 37°C for 3 weeks in the dark. The brains were then dissected, embedded in 3% agarose, and cut at a thickness of 80 µm with a vibratome. The sections were mounted in Mowiol, observed, and photographed by using rhodamine filters.

### Collagen co-culture

Collagen assays were performed as previously described [17], using rhombic lip explants from E11.5 and E12.5 mouse embryos cultured either with conditioned medium from Netrin-1 secreting cells or facing Netrin-1-secreting cells [55]. E11.5 explants are enriched in inferior olivary neurons (ION neurons) whereas E12.5 explants are enriched in neurons that form other caudal PCN, in particular the lateral reticular nucleus (LRN neurons) [17]. After 60–70 hours in a 5% CO_2_, 37°C, 95% humidity incubator, collagen assays were fixed for 20 minutes in 4%PF/4% sucrose at 37°C.

### Quantification of the axon outgrowth and fascicles length in collagen assays

Picture acquisition and processing for axon outgrowth quantification were performed has previously described [18]. Quantification of the length of axon fascicles growing out of the rhombic lips explants was performed by measuring the distance between the edge of the explant and the distal extremity of axon fascicles of 20 fascicles of each explant from wt and *TTL* ^−/−^ embryos. Differences between calculated averages were considered significant when *P*<0,05 using a Student's *t*-test.

### Organotypic culture

The rhombencephalon from E12.5 mouse embryos was opened and flattened with the ventricular side down, onto a Millicell membrane filter (Millipore) laying on BME supplemented with 25% horse serum, 2,5% HBSS, 1% glutamine and 1% glucose (all purchased from GIBCO). After 30–72 hours in a 5% CO_2_, 35°C, 95% humidity incubator, rhombencephalons were fixed for 30 minutes with 4% PF.

### Analysis of growth cone shapes upon GFP transfection in organotypic culture

The size of the growth cones has been quantified through the analysis of the area (A) and perimeter (P) of 20 growth cones from wt and *TTL*−/− neurons, as well as the resulting shape factor (4πA/P^2^) then calculated using the Integrity Morphometry analysis option from Metamorph software (Molecular Devices). A shape factor close to 0 indicates a flattened object whereas a value of 1 indicates a perfect circle. The base of the most proximal filopodia was considered as the beginning of the growth cone. Differences between parameters values were considered significant when *P<0,01* using a Student's *t*-test.

### Immunofluorescence and antibodies

After fixation, cultures were permeabilized with 0,1% Triton X-100 and immunofluorescence was performed in phosphate buffered saline (PBS), 1% normal goat serum (NGS), 0,1% Triton X-100 and 0,2 mg/ml sodium azide. The following primary antibodies were used: anti-glu-tubulin (1∶6000; [56]), anti-tyr-tubulin (1∶6000; clone YL1/2; [57]), monoclonal anti-α-tubulin (1∶4000; clone α3a) from the laboratories of D. Job and J. Wehland, rabbit polyclonal anti-GFP (1∶1000; Molecular Probes), mouse monoclonal anti-class III β-tubulin (TUJ1; 1∶4000; Jackson ImmunoResearch), rabbit polyclonal anti-Myosin light chain 2 (1∶100 Cell Signaling Technology) and anti-MyosinIIB heavy chain (1∶100 Sigma). These primary antibodies were revealed using secondary antibodies raised from goat, directed against rat, mouse or rabbit conjugated to Alexa 488 (1∶1000; Molecular probes) or Cy3 (1∶1000; Jackson ImmunoResearch). F-actin staining was performed with rhodamine-conjugated phalloidin (1∶400; Molecular Probes). DAPI (1 µg/ml, Vector) was used to visualize nuclei.

### GFP electroporation, time-lapse experiments and tracking analysis

GFP electroporation of E12.5 rhombic lips was globally performed as previously described [18]. Four pulses of 30 V were applied to the injected hindbrains using a BTX ECM830 electroporator and CUY610 electrodes (Nepa Gene, Chiba, Japan). Time-lapse experiments were performed using an inverted Zeiss microscope and a CoolSnap *HQ* camera (Roper Scientific). Images were acquired from the second day of culture, at a rate of one image every 5 or 10 minutes, either with a 20× or a 40× long range objective. For quantification of their trajectories straightness and growth speed, neurite tips were tracked using the isosurface and tracking options from Imaris software (Bitplane AG).

### Deconvolution

When necessary, pictures were acquired on a motorised microscope (Leica DMRA2) equipped for image deconvolution. Acquisition was performed using an oil immersion objective (×100 PL APO HCX, 1.4 NA) and a CoolSnap HQ camera. The Z-positioning was accomplished by a piezo-electric motor (LVDT, Physik Instruments). Z-series of images were taken at 0,2 µm increments and the system was steered by the Metamorph Software. Deconvolution was performed by the 3D deconvolution module from Metamorph using the fast iterative Constrained PSF-based algorithm [58].

### Detection of active Rho GTPases and Western blots

Lysates were prepared from E12.5 total hindbrains and then processed as previously described [18]. GST (glutathione S-transferase)-RBD (Rho binding domain)- PAK construct was provided by M. A. Schwartz (State University of New York) and prepared as described in Li et al. [59].

## Supporting Information

Figure S1 TTL−/− PCN neurons lack tyr-tubulin. (A) Immuno-labeling with anti-tyr-tubulin antibody in wt PCN dissociated neurons at E12.5 showed a staining all along the axon, until the distal tip. (B) Merge of tyr-tubulin and glu-tubulin staining revealed that MTs in the distal tip of the axon were only composed of tyr-tubulin since glu-tubulin was absent in this region. Conversely, tyr-tubulin immuno-labeling only showed a faint perinuclear staining in TTL−/− neurons (C) whereas glu-tubulin staining in these neurons was observed all along the neuron, including the distal tip of the axon as shown in merge of tyr- and glu-tubulin immuno-labeling (D). Compared tyr-tubulin proteic levels in hindbrain extracts from two wt and two TTL−/− E12.5 embryos after Western blotting with the anti-tyr-tubulin antibody (E).
(2.95 MB PDF)

Figure S2 Comparison of the morphology of GFP-electroporated growth cones at disinct steps of the PCN migratory pathway. (A) schematic representation of organotypic culture of a GFP-electroporated hindbrain. 1, 2 and 3 indicates the distinct steps of the migratory pathway shown in B and C, D and E, F and G, respectively. (B–E) After 30 h in culture, most of PCN leading processes are still navigating at mid-term between the ipsilateral rhombic lip and the midline (B, C; step 1) while some of them are already reaching it (dashed line) (D, E: step 2). (F, G) Later on, after 3DIV, some leading processes can be seen approaching the contralateral rhombic lip (step 3). At each step, white arrow heads indicate growth cones with hypertrophic morphology or lacking an obvious direction. These growth cones are more frequently observed in TTL−/− hinbrains, but especially when leading processes are getting close to the floor plate (E). Scale bar: 30 µm in B (for B–G).
(2.54 MB PDF)

Figure S3 Comparison of neurites branching in wt and TTL−/− neurons growing in a collagen matrix. The aspect of GFP-electroporated growing neurites from wt (A, B) and TTL−/− (C, D) E12 explants toward Netrin-1 were observed after immuno-labeling with anti-GFP antibody. In wt, neurons extend straight neurites toward Netrin-1 without branching, neither proximally to the cell body nor distally along the neuritic extension (arrows in A, B) whereas in TTL−/−, branches are observed distally along the neurite, or close to the cell body where the leading process initiates (white arrows in C, D). Scale bar in A: 125 µm
(0.56 MB PDF)

Figure S4 Analysis of the effect of increasing concentrations of Rac1 inhibitor on leading process outgrowth and nuclear migration- E12.5 wt rhombic lip explants were cultured in collagen matrix in presence of a range of concentrations of Rac1 inhibitor : 0 mM (A), 2 mM (B), 5 mM (C), 10 mM (D). Axon outgrowth and nuclear migration was then analyzed after anti-total a-tubulin and DAPI immunostainings. Quantifications were obtained using the threshold technique and Metamorph analysis (E). No significant difference could be observed between control condition (n = 12) and 2 mM Rac1 inhibitor treated explants (n = 18), neither for leading process outgrowth nor for nucleokinesis. (C) At 5 mM of Rac1 inhibitor, the outgrowth was significantly decreased but nuclear migration was not affected (n = 18). (D) At the highest concentration, 10 mM, both axon outgrowth and nuclear migration were significantly impaired compared to control conditions (n = 18). ** P<0,001. Scale bar : 500 µm.
(0.75 MB PDF)

Movie S1 (13.07 MB AVI)

Movie S2 (10.69 MB AVI)

Movie S3 Movie S3 shows a wt PCN neuron getting out of the rhombic lip explant and entering easily in the collagen matrix toward the Netrin-1 source.
(2.48 MB AVI)

Movie S4 Movie S4 shows a TTL−/− PCN neuron at the border of rhombic lip explant and failing to enter the collagen matrix to further grow toward the Netrin-1 source. Instead, the neuron emit numerous primary and secondary neurites that explore the edge of the explant and never succeed in going past it, finally resulting in change of its migration direction.
(41.48 MB AVI)
