# Supplementary figures and images for: Tubulin Tyrosination Is Required for the Proper Organization and Pathfinding of the Growth Cone
Source: PLoS One. 2009 Apr 30;4(4):e5405. doi: 10.1371/journal.pone.0005405 (PMC2672595; doi:10.1371/journal.pone.0005405)

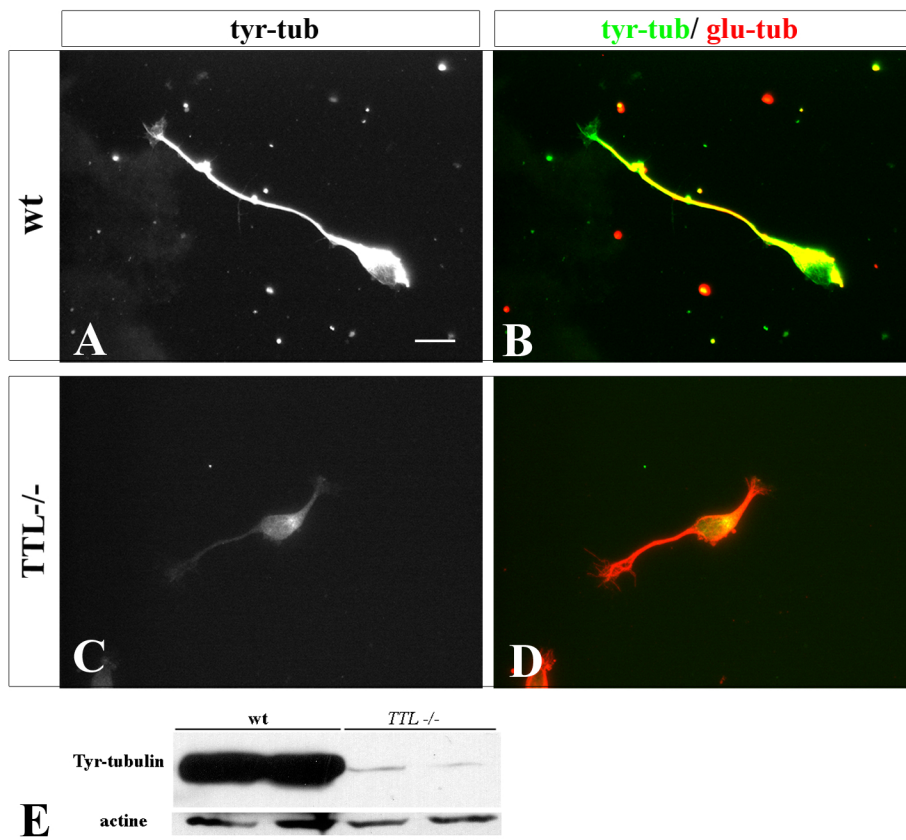

**Supplemental Figure S1**

Supplement: Figure S1 — TTL−/− PCN neurons lack tyr-tubulin. (A) Immuno-labeling with anti-tyr-tubulin antibody in wt PCN dissociated neurons at E12.5 showed a staining all along the axon, until the distal tip. (B) Merge of tyr-tubulin and glu-tubulin staining revealed that MTs in the distal tip of the axon were only composed of tyr-tubulin since glu-tubulin was absent in this region. Conversely, tyr-tubulin immuno-labeling only showed a faint perinuclear staining in TTL−/− neurons (C) whereas glu-tubulin staining in these neurons was observed all along the neuron, including the distal tip of the axon as shown in merge of tyr- and glu-tubulin immuno-labeling (D). Compared tyr-tubulin proteic levels in hindbrain extracts from two wt and two TTL−/− E12.5 embryos after Western blotting with the anti-tyr-tubulin antibody (E). (2.95 MB PDF) [file pone.0005405.s001.pdf]

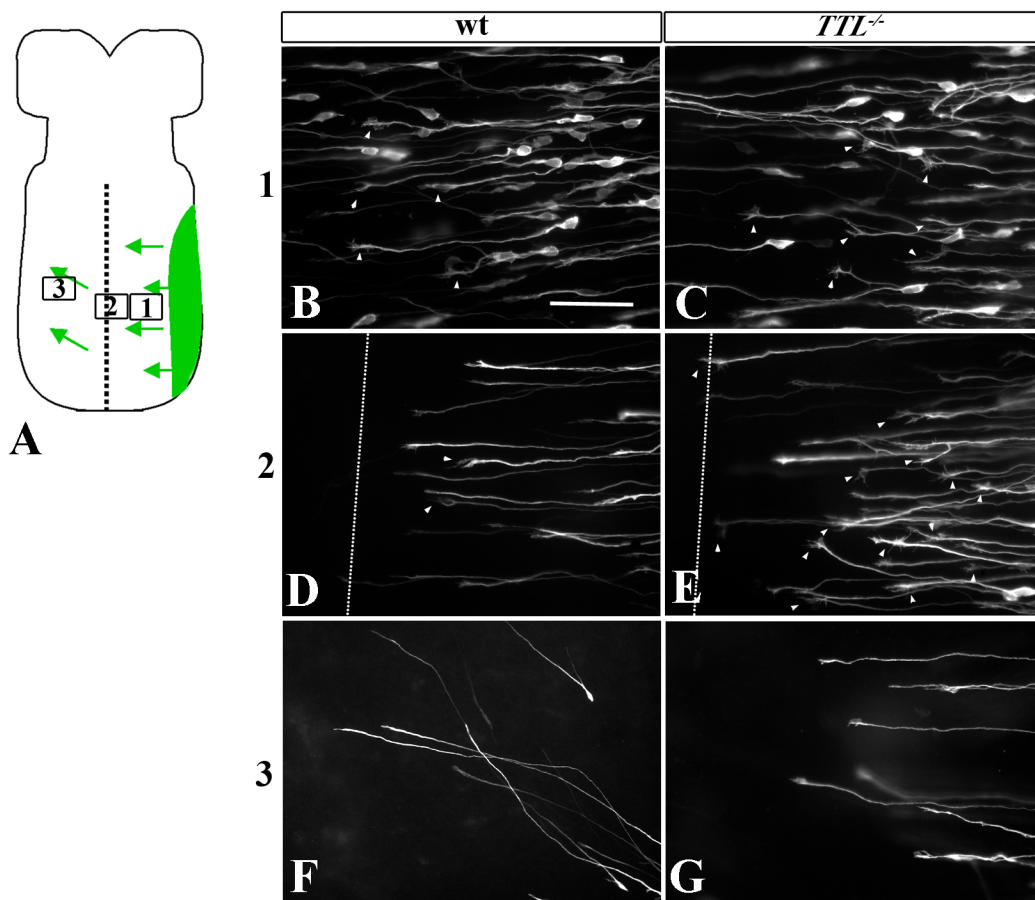

**Supplemental Figure S2**

Supplement: Figure S2 — Comparison of the morphology of GFP-electroporated growth cones at disinct steps of the PCN migratory pathway. (A) schematic representation of organotypic culture of a GFP-electroporated hindbrain. 1, 2 and 3 indicates the distinct steps of the migratory pathway shown in B and C, D and E, F and G, respectively. (B–E) After 30 h in culture, most of PCN leading processes are still navigating at mid-term between the ipsilateral rhombic lip and the midline (B, C; step 1) while some of them are already reaching it (dashed line) (D, E: step 2). (F, G) Later on, after 3DIV, some leading processes can be seen approaching the contralateral rhombic lip (step 3). At each step, white arrow heads indicate growth cones with hypertrophic morphology or lacking an obvious direction. These growth cones are more frequently observed in TTL−/− hinbrains, but especially when leading processes are getting close to the floor plate (E). Scale bar: 30 µm in B (for B–G). (2.54 MB PDF) [file pone.0005405.s002.pdf]

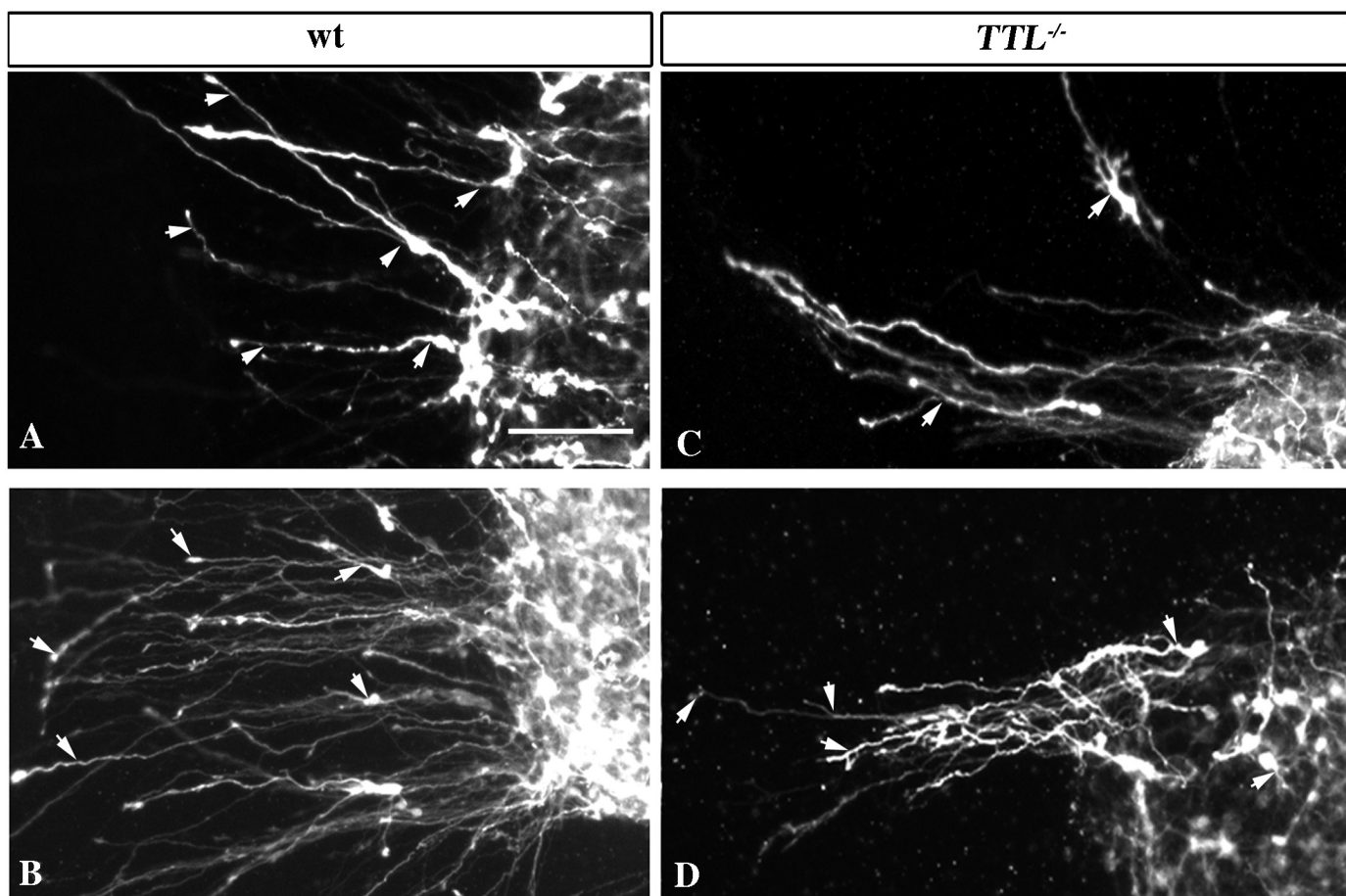

**Supplemental Figure S3**

Supplement: Figure S3 — Comparison of neurites branching in wt and TTL−/− neurons growing in a collagen matrix. The aspect of GFP-electroporated growing neurites from wt (A, B) and TTL−/− (C, D) E12 explants toward Netrin-1 were observed after immuno-labeling with anti-GFP antibody. In wt, neurons extend straight neurites toward Netrin-1 without branching, neither proximally to the cell body nor distally along the neuritic extension (arrows in A, B) whereas in TTL−/−, branches are observed distally along the neurite, or close to the cell body where the leading process initiates (white arrows in C, D). Scale bar in A: 125 µm (0.56 MB PDF) [file pone.0005405.s003.pdf]

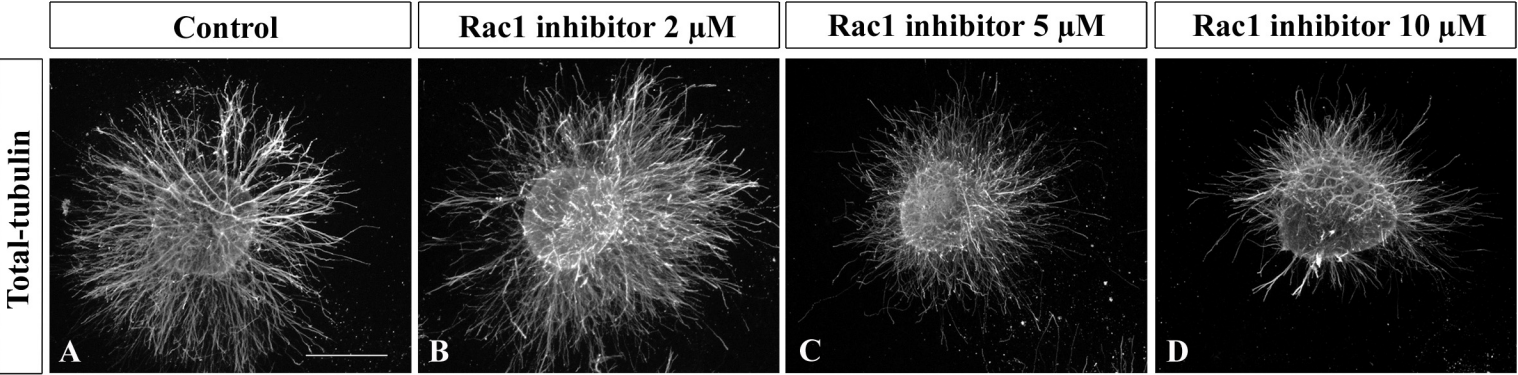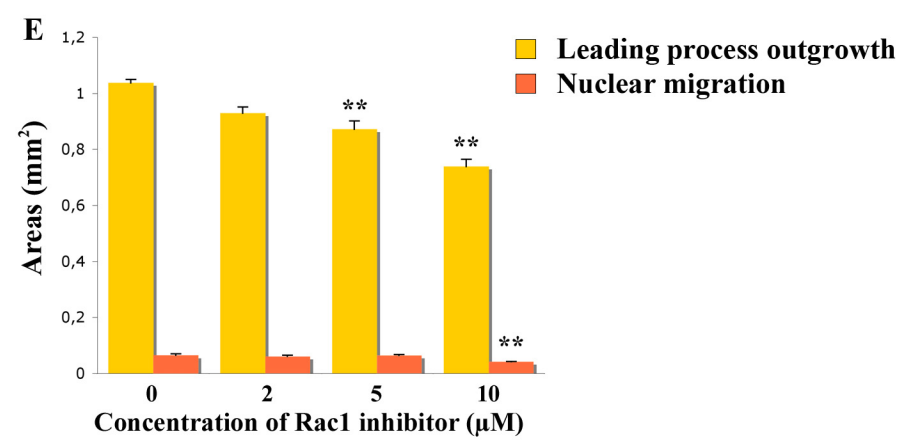

**Supplemental Figure S4**

Supplement: Figure S4 — Analysis of the effect of increasing concentrations of Rac1 inhibitor on leading process outgrowth and nuclear migration- E12.5 wt rhombic lip explants were cultured in collagen matrix in presence of a range of concentrations of Rac1 inhibitor : 0 mM (A), 2 mM (B), 5 mM (C), 10 mM (D). Axon outgrowth and nuclear migration was then analyzed after anti-total a-tubulin and DAPI immunostainings. Quantifications were obtained using the threshold technique and Metamorph analysis (E). No significant difference could be observed between control condition (n = 12) and 2 mM Rac1 inhibitor treated explants (n = 18), neither for leading process outgrowth nor for nucleokinesis. (C) At 5 mM of Rac1 inhibitor, the outgrowth was significantly decreased but nuclear migration was not affected (n = 18). (D) At the highest concentration, 10 mM, both axon outgrowth and nuclear migration were significantly impaired compared to control conditions (n = 18). ** P<0,001. Scale bar : 500 µm. (0.75 MB PDF) [file pone.0005405.s004.pdf]
